# The spatio-temporal pattern and its influencing factors of production efficiency of health resources in China

**DOI:** 10.3389/fpubh.2024.1376518

**Published:** 2024-04-16

**Authors:** Hui Tang, Rongjun Ao, Yilei Li

**Affiliations:** ^1^College of Architecture and Urban Planning, Hunan City University, Yiyang, Hunan, China; ^2^College of Urban and Environmental Sciences, Central China Normal University, Wuhan, Hubei, China; ^3^Key Laboratory of Key Technologies of Digital Urban-Rural Spatial Planning of Hunan Province, Yiyang, Hunan, China; ^4^College of Urban and Environmental Sciences, Hunan University of Technology, Zhuzhou, China

**Keywords:** health productivity of health resources, spatial and temporal patterns, influencing factors, prefecture-level regions, China

## Abstract

There is always a contradiction between the limited health resources and the unlimited demand of the population for health services, and only by improving the productivity of health resources can the health level of the population be improved as much as possible. Using prefecture-level administrative regions as spatial units, the paper analyzes the spatial pattern and changes of health productivity of health resources in China from 2000 to 2010, and uses a spatial panel Tobit model to examine the effects of factors such as technical level of health institutions, health service accessibility, public health policies and ecological environment quality on health productivity of health resources. The results show that with the Hu Huanyong line as the dividing line, the spatial heterogeneity of “high in the southeast and low in the northwest” in the health productivity of China's health resources is clear; as the regional differences narrow, the spatial correlation increases, and the spatial pattern of “overall dispersion and partial agglomeration” becomes more obvious. The fitting results of the spatial Durbin model reveal the direction and degree of influence of local and adjacent factors on the production efficiency of health resources. The positive influence of technical level of local health institutions and the accessibility of health services, the literacy level and the ability to pay for health services of residents in adjacent areas, the degree of urbanization of regional health resource allocation, climate suitability and the quality of the atmospheric environment are significant. And the negative influence of local residents' literacy and ability to pay for health services, the technical level of health institutions in adjacent areas and the degree of medicalization of health resource allocation are also significant. The influence of the degree of medicalization of local health resource allocation and the accessibility of health services in adjacent areas are significantly spatial-heterogeneous.

## 1 Introduction

Health resources are the basis of medical and health services, and are essential for maintaining and improving national health. As the level of economic development has increased, China's medical and health care has developed rapidly and the scale of health resource supply has continued to expand. The national per capita health expenditure increased from 361.9 yuan in 2000 to 5,112 yuan in 2020, the number of health technicians per 1,000 people increased from 3.63 to 7.57, and the number of beds in medical institutions per 1,000 people increased from 2.47 to 6.45. However, health resources are always scarce compared to the growing health needs of the population. The Outline of the National Healthcare Service System Plan (2015–2020) states that the problems of insufficient total health resources and unreasonable structure and layout are still prominent in China; the outline of the “Healthy China 2030” plan also points out that the contradiction between the overall shortage of health service supply and the growing demand is still prominent. As China's social economy enters a new normal, the supply-side structural reform of the health care system is being promoted in depth, and optimizing the allocation of health resources rather than just increasing the supply of health resources is the basic policy direction. Therefore, under the constraint of limited resources, only by improving the productivity of health resources and maximizing the level of health output can we maximize the promotion of population health improvement and health equity development. China is a vast country, and there are still significant regional differences in health resource endowment and residents' health level. The interregional comparative study of health productivity of health resources reveals the influence of exogenous controllable factors such as ecological environment, socio-economic and medical policies on health productivity of health resources, which can provide theoretical basis for the decision to optimize the spatial allocation of health resources and improve the efficiency of health resource utilization.

Nunamaker (1), Sherman (2), and Banker et al. (3), based on Farrell's (4) seminal study of productivity, were early adopters of data envelopment analysis (DEA) to measure the productivity of health care organizations, and health productivity has thus become a research hotspot in areas such as public health management and health eco-nomics. Overall, microscopic studies with health care organizations as the decision making unit still dominate the research in this field, and some of them reveal the influence of factors such as the attribute characteristics of health care organizations (5–8), the market characteristics of health care services (9, 10), and health care policies (11–13) on the operational efficiency of health care organizations. In the mid-to-late 1990s, macro studies with national or regional policy making units proliferated, stemming from a focus on health production efficiency in OECD countries. Färe et al. (14) examined the changes in health productivity in 19 OECD countries from 1974 to 1989 using the DEA-Malmquist index method and concluded that technological progress was the main source of health productivity growth in 10 of these countries. More recently, Mohamadi et al. (15) used the DEA-Malmquist index to assess changes in health productivity in upper middle-income countries in the world from 2009 to 2015, proposing to improve the efficiency of their health care systems by exploring the root causes of internal inefficiencies. Henriques and Gouveia (16) used the Value-Based DEA model to assess the impact of the COVID-19 epidemic on the efficiency of state-owned hospitals in Portugal and found that inefficiencies in the public health sector were not due to a lack of health resources, but rather to inefficiencies in their use. Subsequently, many studies with countries as the decision-making unit have revealed the influence of factors such as socioeconomic (17, 18) and behavioral characteristics of the population (19), health care systems and policies (9, 20, 21), and living conditions and the environment of the place of residence (18, 22) on health productivity.

Many domestic studies take provincial-level administrative regions as the decision-making unit, while a small number of studies targeting provincial areas take prefectural or county-level administrative regions as the decision-making unit to explore the factors influencing regional health production efficiency and changes. Factors affecting regional differences in health productivity include accessibility of health services (23), residents' ability to utilize health services (23), marketability of health services (24), management level of health care institutions (25), level of economic development (26), regional urbanization rate (26, 27), and health care policies (28). It has also been found that the increase in the share of for-profit and high-quality hospitals is favorable to the technical efficiency of hospitals, and government subsidies are not favorable to the technical efficiency of hospitals in coastal provinces and districts (29). Economic growth, total population and structure significantly affect the combined technical efficiency of provincial health resources in China from 2013 to 2016 (30). The increase in the population's education level has a positive effect on health productivity, while the percentage of health insurance participation has a negative effect on health productivity (26). Other studies such as Xiang et al. (27) used a spatial panel Tobit model to reveal the significant effects of total population urbanization rate, education funding, and aging rate and the number of public hospitals on the efficiency and changes in provincial health resource allocation in China before and after health care reform. Xia et al. (28) used a three-stage data envelope model to analyze the effects of economic development, population density, policy support, social base, and institutional operations on the efficiency of provincial health resource allocation in China from 2009-2018. Some studies have also revealed a decline in health production efficiency in most Chinese provinces and regions after 2009, arguing that optimizing resource allocation, for example, can help improve health production efficiency (31). In contrast, in a study using county level administrative regions as the decision-making unit, Yang et al. (32) studied intercounty differences in health productivity in Hubei Province from 2012 to 2014 and concluded that regional economic development, household income of residents, and local medical policies significantly affected county health productivity; Wang et al. (33) concluded that supply demand mismatch led to inefficient allocation of health re-sources in Chongqing counties during 2009–2017.

Domestic and international academic research on health productivity of health resources has yielded fruitful results, and a certain consensus has been reached on the measurement techniques and exploration of influencing factors of health productivity. However, research in this area is still largely focused on the public health management and health economics communities, and the absence of geography has led to the following deficiencies in current research: First, spatial pattern analysis is lacking. The analysis of spatial pattern and change process is the basis for revealing the spatial and temporal patterns of health productivity of health resources and providing solid empirical evidence to support the study of influencing factors. Second, the spatial scale is too large. Using national or provincial areas as spatial units for spatio-temporal process and influence factor studies, the influence of heterogeneous factors within regions on spatial pattern analysis and inter-regional comparative studies cannot be eliminated, which may lead to biased research results. Third, the study of influence factors is in-sufficient. The empirical analysis of influence factors ignores the influence of spatial factors, which may lead to biased empirical results. While the influencing factors primarily focus on health care institutions, there is insufficient consideration of exogenous controllable factors that may impact the efficiency of health production in regional health resources.

## 2 Materials and methods

### 2.1 Theoretical framework

**Figure 1:**
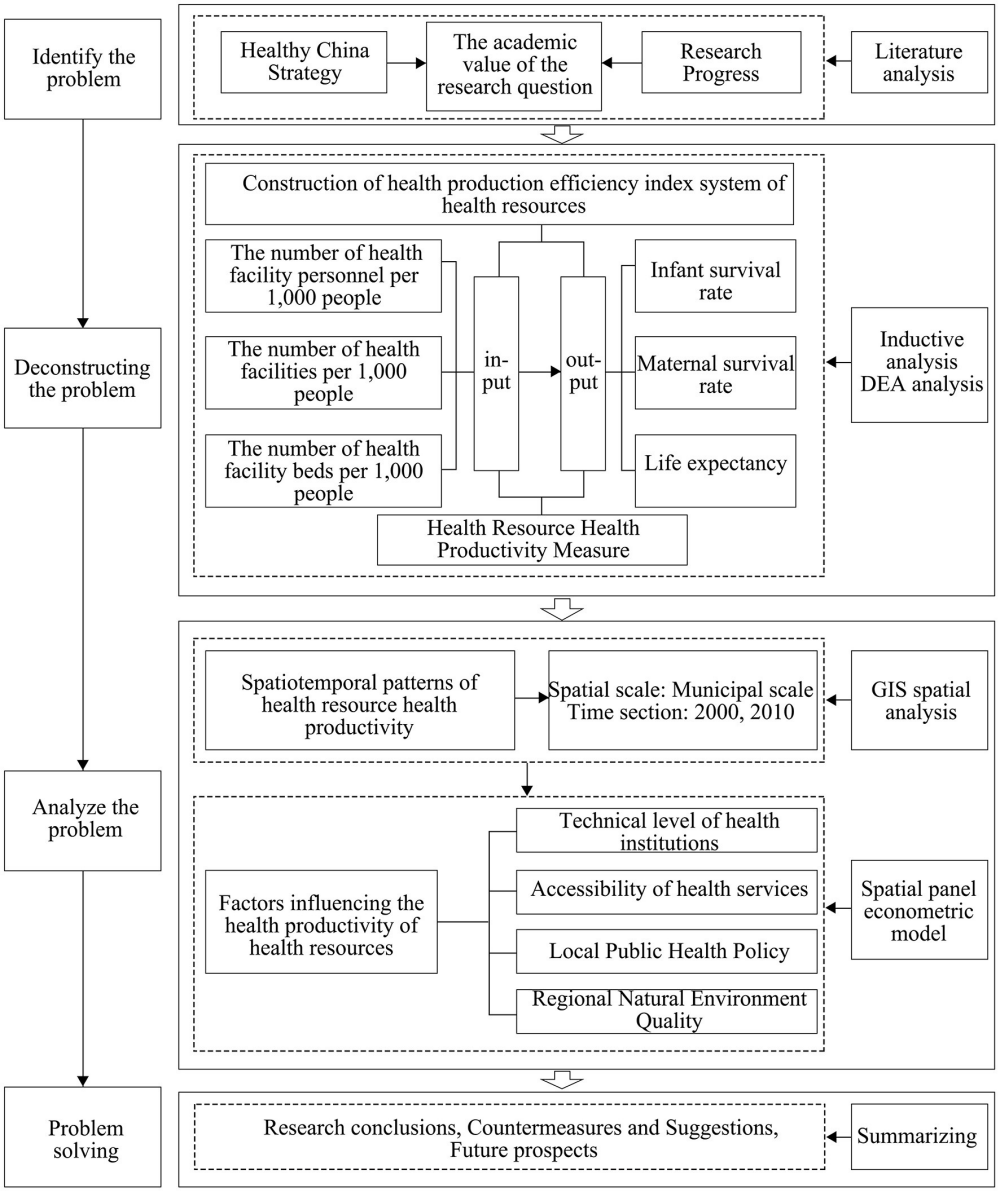
Theoretical framework.

The regional health system can be regarded as a production system that converts health resource inputs into health outputs, and the regional health resource health production efficiency can be defined as the efficiency of the health system in converting health resource inputs into health outputs. Increases in the level of health output are constrained by a combination of the amount of health inputs and the productivity of health. Therefore, pursuing only an increase in health inputs while neglecting the improvement of health productivity may result in a waste of health resources and may not necessarily lead to an effective increase in the level of health outputs (29). By constructing an indicator system of regional health resource inputs and outputs and measuring regional health resource health production efficiency, we further analyze the spatial and temporal patterns and changing characteristics of regional health resource health production efficiency in China. From the perspective of system theory, the health resources of the region are compared to the main body of the health system, the residents of the region are compared to the objects of the health system, and the inputs of health resources and the outputs of the health level of the residents are regarded as the inputs and outputs of the health service system. The influences on the health productivity of regional health resources can be interpreted from both the internal and external aspects of the system (34). From within the system, it is clear that the level of management and technology of health organizations is a direct factor affecting the efficiency of the health production of health resources, given that the total amount of health resources invested in the region remains unchanged. From the external point of view of the system, the degree of difficulty in realizing the health services provided by the main actors of the system for the target population will have a direct impact on the efficiency of the health output of the health resources, while the natural environment is a baseline factor affecting the health of the population. Therefore, based on theoretical analysis and current research results, this paper intends to analyze its impact on the health productivity of regional health resources from four aspects, namely, the technical level of health institutions, accessibility of health services, local public health policies and Regional Natural Environment Quality (Figure 1).

### 2.2 Study area

The study area is mainland China, and Hong Kong, Macao, Taiwan and the South China Sea islands are not included in the study. Because the health productivity measurement indicators of life expectancy to the prefecture-level region can only be temporarily to 2010, the time period of this study is determined as 2000–2010, limited to the availability of data, mainly examine the two node years of 2000 and 2010. The spatial unit is the prefecture-level administrative district (hereinafter referred to as the prefecture-level region), mainly based on two considerations: (1) Within the framework of the general layout of health resources, which involves a gradient allocation based on territorial levels, the prefecture-level administrative regions are the most important administrative units for the spatial allocation standards of health resources and the implementation of regional health planning in China. The National Healthcare Service System Planning Outline (2015–2020) clearly states that the provincial government is responsible for setting standards for the allocation of health resources and refining bed allocation standards to local municipalities. Local municipal governments, on the other hand, are responsible for preparing and implementing regional health plans and medical institution setting plans. They also coordinate the planning of various types of medical and health institution settings within local municipalities in accordance with the principle of territoriality. Therefore, using prefecture-level administrative regions as the spatial unit for empirical analysis can enhance the reference and guidance of this study on the spatial allocation of health resources. (2) Balancing theoretical requirements and practical constraints. In order to eliminate the influence of the non-homogeneity of the geographical environment in the region on the inter-regional comparative study, the spatial scale of the empirical analysis should be as small as possible. However, if the spatial scale is too small, essential data may not be available. At present, the smallest spatial unit for which basic data can be obtained more completely is the prefecture-level administrative district.

In order to ensure the dynamic comparability of spatial data, according to the information of administrative division changes provided by the administrative division network (www.xzqh.org), the prefecture-level administrative divisions of all years were uniformly adjusted according to the caliber of 2010, and the four municipalities directly under the central government and the directly administered county-level administrative divisions of some provinces were treated as spatial units alongside with the prefecture-level regions, forming a total of 341 spatial units.

### 2.3 Methods

#### 2.3.1 Health productivity measure

Accordingly, each region can be considered as a decision unit of health production, and the DEA method can be used to measure the efficiency of health production of health resources in each region. Among them, the input variables of health production are measured by regional health resource endowment, including human resources, physical resources, and financial resources, and are quantified by three variables: the number of health facility personnel per 1,000 people, the number of health facilities per 1,000 people, and the number of health facility beds per 1,000 people. The output variables are measured by the average health status of the regional population, and positive indicators such as infant survival rate, maternal survival rate and life expectancy are measured according to the main indicators of health construction set in the “Healthy China 2030” plan outline. The evaluation model uses the output-oriented BCC model based on the following considerations: first, health resources are scarce resources, and regional health care systems should generally maximize health output with limited inputs, thus adopting output-oriented production efficiency. Second, health production in regional health systems is often not at an optimal scale, and health output can thus be viewed as an increasing function of health inputs (20). The BCC model assumes variable payoffs for scale of production of decision units, which is more consistent with the reality of health production in regional health care systems (35). Under variable payoffs to scale, the combined efficiency of output-oriented health production can be decomposed into pure technical efficiency and scale efficiency. The former reflects the change in regional health output due to factors such as management and technology under certain input scale conditions; the latter reflects the difference between the actual production scale of regional health resources and the optimal production scale under certain management and technology conditions.

#### 2.3.2 Spatial autocorrelation analysis

Based on ArcGIS software platform, we statistically analyzed the spatial association patterns of health resource productivity at the prefecture level in China. Through interannual comparisons, we analyze the change process of spatial association patterns, summarize the general rules of spatial and temporal processes of health resource health production efficiency, and provide empirical evidence support for the study of influencing factors. In this regard, the region-wide spatial autocorrelation portrays the spatial correlation pattern of health productivity over the study area as a whole, using the region-wide Moran index *I* as the statistic, calculated as Equation 1:


(1) $$\begin{array}{l} I=\frac{\sum_{i=1}^{n}\sum_{j=1}^{n}W_{ij}\left(y_{i}-ȳ\right)\left(y_{j}-ȳ\right)}{\sigma \sum_{i=1}^{n}\sum_{j=1}^{n}W_{ij}} \end{array}$$

where *i* and *j* represent regions, *y* represents health productivity of health resources, *n* is the number of regions, ȳ is the mean of health productivity, *f* is the variance of health productivity, and *W*_*ij*_ is the spatial weight matrix. The values of *I* range from [−1, 1], and when significantly positive, the inter-regional distribution of health productivity is positively correlated; when significantly negative, the inter-regional distribution of health productivity is negatively correlated; and when equal to 0, health productivity is randomly distributed in the whole area. Local spatial autocorrelation analysis identifies patterns of association between the health productivity of each region and other regions, as reflected by the local Moran index *I*_*i*_, calculated as Equation 2:


(2) $$\begin{array}{l} I_{i}=\frac{y_{i}-ȳ}{\sigma }\sum_{j=1}^{n}W_{ij}\left(y_{j}-ȳ\right) \end{array}$$

If *I*_*i*_ > 0 and is significant, it means that region i is positively correlated with the health productivity of other regions. If *I*_*i*_ < 0 and is significant, it means that region i is negatively correlated with the health productivity of other regions. If *I*_*i*_ = 0, region *i* is not correlated with the health productivity of other regions. The spatial weight matrix measures the degree of association between regions.

#### 2.3.3 Spatial divergence analysis

Spatial heterogeneity testing is a necessary part of spatial data analysis (36), and should first detect the presence of spatial heterogeneity to determine whether to use a global or local model. Geographic probes were used to analyze the spatially stratified heterogeneity of health productivity of health resources. *q* The expression for the statistic is Equation 3:


(3) $$\begin{array}{l} q=1-\frac{1}{N\sigma^{2}}\overset{L}{\underset{h=1}{\sum }}N_{h}\sigma_{h}^{2} \end{array}$$

where *h* is the stratification of health resource health productivity, *L* is the number of strata, *N*_*h*_ and *N* are the number of samples of the terrestrial regions in stratum h and the whole domain, respectively, and $\sigma_{h}^{2}$ and σ^2^ are the variances of health resource health productivity in stratum *h* and the whole domain, respectively. *q* has a value range of [0, 1], and larger values indicate more pronounced spatial heterogeneity.

#### 2.3.4 Spatial panel Tobit model

The direction and degree of influence of each factor variable on the overall efficiency of health production was analyzed empirically using multiple regression methods with the overall efficiency of health production of health resources as the explanatory variable. Since health productivity is truncated data, a restricted dependent variable model (Tobit model) was selected and a panel data consisting of 341 prefecture-level regions from 2000 to 2010 was used to fit the model. Considering the existence of spatial effects, a spatial econometric model was used. The general form of the spatial panel data model is Equation 4:


(4) $$\{\begin{array}{l} eff_{it}=\rho W_{ij}eff_{it}+\sum_{r}\left(\beta_{r}X_{it}^{r}\right)+\sum_{r}\left(\theta_{r}W_{ij}X_{it}^{r}\right)+\mu_{i}+\upsilon_{t}+\varepsilon_{it}\\ \varepsilon_{it}=\lambda W_{ij}\varepsilon_{it}+\varphi_{it} \end{array}\text{​​​​​​​​}$$

where *i* and *j* represent regions, *t* represents years, *eff* represents health resource health productivity, and *X* represents a set of variables that affect health productivity. *W* is a spatial weight matrix. ρ is the coefficient of the spatial lag term of health productivity, which reflects the influence of health productivity in other regions on local health productivity; β is the regression coefficient of the influencing factors. θ is the coefficient of spatial lag term of influence factors, which reflects the influence of influence factors in other regions on local health productivity; λ is the coefficient of spatial error term of health productivity, which reflects the influence of error shocks to health productivity in other regions on local health productivity. μ is the individual effect, υ is the time effect, and ε and φ are random perturbation terms. The above model is a spatial Durbin model (SDM) if λ = 0 and a spatial lagged model (SLM) if λ = 0 and θ = 0. If ρ = 0 and θ = 0, the spatial error model (SEM) is used. The Lagrange multiplier test was applied to determine whether SLM, SEM or SDM was used, and the Hausman test was applied to determine whether a fixed-effects model or a random-effects model was used.

With reference to existing studies and empirical observations, the factors influencing the health productivity of regional health resources can be attributed to four aspects: technical level of health institutions, health service accessibility, public health policy, and ecological quality (Table 1).

1. Technical level of health institutions. The technical level of health institutions is a direct factor affecting the health productivity of health resources, given that the total amount of regional health resources input remains unchanged. The number of health technicians as a share of the number of health institution personnel is used to measure the technical level of health institutions.
2. Accessibility of health services. Generally, improved resident access to health services enhances the likelihood of maintaining and improving health, facilitating more efficient transformation of health resources into outputs, assuming a constant health resource endowment. However, heightened accessibility may also lead to over-consumption, potentially resulting in a mismatch between improved health status and resource consumption. This could even have a crowding-out effect on reasonable health demand, potentially reducing the health productivity of resources. The accessibility of health services is reflected in three aspects: first, the accessibility of regional health services. Accessibility is the basic factor of health service accessibility, while the coverage of health institutions to residents and the accessibility of transportation are the main representations of regional health service accessibility. The health service accessibility index, calculated by five indicators: population density, density of health institutions, density of graded roads, per capita urban road area and the number of buses owned by 10,000 people, is a comprehensive measure of the accessibility of regional health services. Second, the residents' perceptions of health services. The level of awareness of health and health services affects residents' health resource utilization decisions (37, 38), while the level of education (39, 40) and socio-cultural prevalence are important factors that influence the level of awareness of health services. The health service awareness index is a comprehensive measure of regional residents' awareness of health services, calculated using three indicators: the share of the population with university education and above in the population aged 6 and above, the average number of years of education, and the number of books in public libraries per capita. Income level directly determines the residents' ability to pay for health services. Residents with high income levels have a high purchasing power for health services and a high likelihood of accessing health services. The ability to pay index, calculated by three indicators: the average wage of urban workers on the job, urban per capita disposable income and rural per capita disposable income, is a comprehensive measure of the ability of regional residents to pay for health services. The basic idea of the calculation of the above three indices is: first, standardize the value of the measurement index; then, in order to eliminate the subjective preference of the index assignment, use the entropy value method to determine the weight of each index; finally, weight the standardized value of each index and take the average value to get the index value.
3. Local public health policies. Health care services are typically public services, and government policies on health resource allocation and management can directly affect the productivity of the health care delivery system. First, the degree of medicalization of health resource allocation. Residents' health maintenance and improvement require not only disease treatment services, but also disease prevention services. If health resources are highly concentrated on medical services, and disease prevention and prevention resources are neglected to be equipped, the health risk of the population will increase, which is not conducive to health productivity improvement (41). Thus, the structure of health resource endowment is an important influence on the efficiency of health resource health production. The degree of medicalization of health resource allocation is measured by the share of the number of physicians in regional hospitals in the number of health facility personnel. The second is the degree of urbanization of health resource allocation, which refers to the degree of concentration of health resources to cities. Excessive concentration of health resources in cities, particularly large ones, may lead to a supply exceeding demand, resulting in inefficient use of health resources (41). The degree of urbanization of health resource allocation is measured using the ratio of the number of hospital beds per capita in the municipal district to the number of hospital beds per capita in the region.
4. Quality of the natural environment of the region. The natural environment is a basal factor affecting the health of the population. A suitable natural environment has a positive effect on controlling human biological rhythms and enhancing immune function (42), while environmental pollution can cause short- or long-term health damage to the human body. The quality of the natural environment thus becomes an important factor affecting the health productivity of health resources. The efficiency of health output of health services may vary between regions due to differences in the quality of the natural environment, even though health resource inputs and accessibility are the same. The quality of a region's natural environment is measured in two ways. One is climate suitability, measured by the temperature and humidity index, which is calculated as *HI* = (1.8*t*+32)−0.55(1−*f*)·(1.8*t*−26 ). Where *t* stands for the average annual temperature (in degrees Celsius) and *f* stands for the average annual relative air humidity. A very comfortable climate with a temperature-humidity index value of 60–65, as defined by Feng et al. (43) and others; the temperature and humidity index is < 60 or more than 65, the climate is gradually uncomfortable, and the smaller or larger the value, the more uncomfortable the climate. In order to match the temperature and humidity index from small to large with the climate suitability from high to low, the value of the humidity and heat index is treated as follows: if 60 ≤ *HI* ≤ 65, *HI* takes the value 0; for *HI* < 60, let *HI* = |60−*HI*|; *HI*>65, let *HI* = |65−*HI*|. Second, air pollution, measured by industrial sulfur dioxide emissions per square kilometer.

**Table 1:** Influencing factors and their measure indices of production efficiency of health resources.

**Influencing factors**	**Variables**	**Symbols**	**Measurements**	**2000; mean/STD**	**2010; mean/STD**
Technical level of health institutions	Technical level of health institutions	*TL*	Number of health technicians as a share of the number of health institution personnel (%)	0.779/0.087	0.802/0.079
Accessibility of health services	Accessibility of district health services	*HA*	Regional Health Service Accessibility Index	0.014/0.010	0.026/0.018
	Awareness level of residents' health services	*HC*	Index of the level of awareness of health services among regional residents	0.019/0.023	0.027/0.022
	Ability to pay for residential health services	*HP*	Regional residents' ability to pay for health services index	0.020/0.011	0.098/0.029
Local public health policy	Medicalization of health resource allocation	*LT*	Number of doctors in regional hospitals as a share of the number of personnel in health institutions (%)	0.459/0.082	0.410/0.050
	Urbanization of health resource allocation	*LU*	Ratio of hospital beds per capita in municipalities to regions (%)	2.215/1.171	2.140/3.303
Regional natural Environment quality	Climate suitability	*HI*	Temperature and humidity index	0.126/0.081	0.124/0.082
	Atmospheric pollution	*AP*	Industrial sulfur dioxide emissions per square kilometer (WT/KM^2^)	2.869/5.852	0.570/0.779

### 2.4 Data

The basic data are mainly from “China 2000 Population Census Sub-county Information”, “China 2010 Population Census Sub-county Information”, “China Regional Economic Statistical Yearbook”, “China Urban Statistical Yearbook”, as well as China's provincial and regional statistical yearbooks, health and family planning yearbooks (health and health yearbooks), etc. Life expectancy data for the regional population is calculated using the life table method. Data on infant mortality and maternal mortality are difficult to obtain, and are mainly collected through provincial and regional yearbooks, health and family planning yearbooks (health yearbooks), statistical bulletins on national economic and social development, government work reports and annual summary reports, as well as statistical monitoring and analysis reports on women's and children's development planning by local municipalities, publicly available data from maternal and child health centers, and the official website of the Women's Federation. A small amount of missing data was filled in by spatial interpolation. The natural geographic environment data were taken from the National Science Data Sharing Project Earth System Science Data Sharing Platform. The map base map was unified using the standard map provided by the website of the National Basic Geographic Information Center [review number GS(2016)1549].

## 3 Results and conclusion

This section may be divided by subheadings to provide a concise and precise description of the experimental results, their interpretation, and the conclusions drawn.

### 3.1 Spatial-temporal pattern of health productivity of health resources

The pure technical efficiency values of health production of health resources in the 341 prefecture-level regions of the country were close to 1 in both 2000 and 2010, with very small differences, and scale efficiency thus dominated the spatial pattern and variation of the overall efficiency (Table 2). This indicates that the changes in health output due to factors such as management and technology are basically consistent across regions under a certain scale of inputs. For the sake of convenience, the spatial pattern and change characteristics of health production efficiency of regional health resources at the prefecture level in China are analyzed using the composite efficiency as the observed indicator. Table 2 shows that there are significant regional differences in the health productivity of health resources in China. The standard deviation of the inter-regional distribution of comprehensive efficiency expanded from 0.149 in 2000 to 0.156 in 2010, indicating that the absolute differences in the health productivity of health resources at the prefecture level in China have gradually expanded. The coefficient of variation narrowed from 0.467 in 2000 to 0.295 in 2010, suggesting that while relative differences remain sizable, they exhibit a decreasing trend, and the spatial distribution tends to be more dispersed.

**Table 2:** Disparities of production efficiency of health resources between prefectural districts in China.

**Indicators**	**Comprehensive efficiency**		**Pure technical efficiency**		**Scale efficiency**	
	**2000**	**2010**	**2000**	**2010**	**2000**	**2010**
Average value	0.319	0.528	0.999	1.000	0.319	0.528
Standard deviation	0.149	0.156	0.001	0.000	0.149	0.156
Extreme difference value	0.960	0.950	0.005	0.003	0.960	0.950
Coefficient of variation	0.467	0.295	0.001	0.000	0.467	0.295

In general, the southeast-northwest divergence of health productivity of health resources at the prefecture level in China is more obvious (Figure 1). Taking the Hu Huan-yong line (hereafter referred to as Hu-line) as the dividing line, the geographical area to the east concentrated 93.10% of the high and second-highest values of health productivity of health resources in 2000 and 78.85% of the high and second-highest values in 2010. In addition, the Hu-line was used as a dividing line to divide the whole area into two layers, and the spatial heterogeneity of the health productivity of health resources was examined with the geodetector *q* statistic. The *q*-values of 0.978 and 0.976 in 2000 and 2010, respectively, and both passed the 0.01 significance level test, verified the spatial heterogeneity of health productivity of health resources in China, indicating that the Hu-line is statistically significant as a dividing line for the large potential of geographical heterogeneity of health productivity of health resources in China.

**Figure 2:**
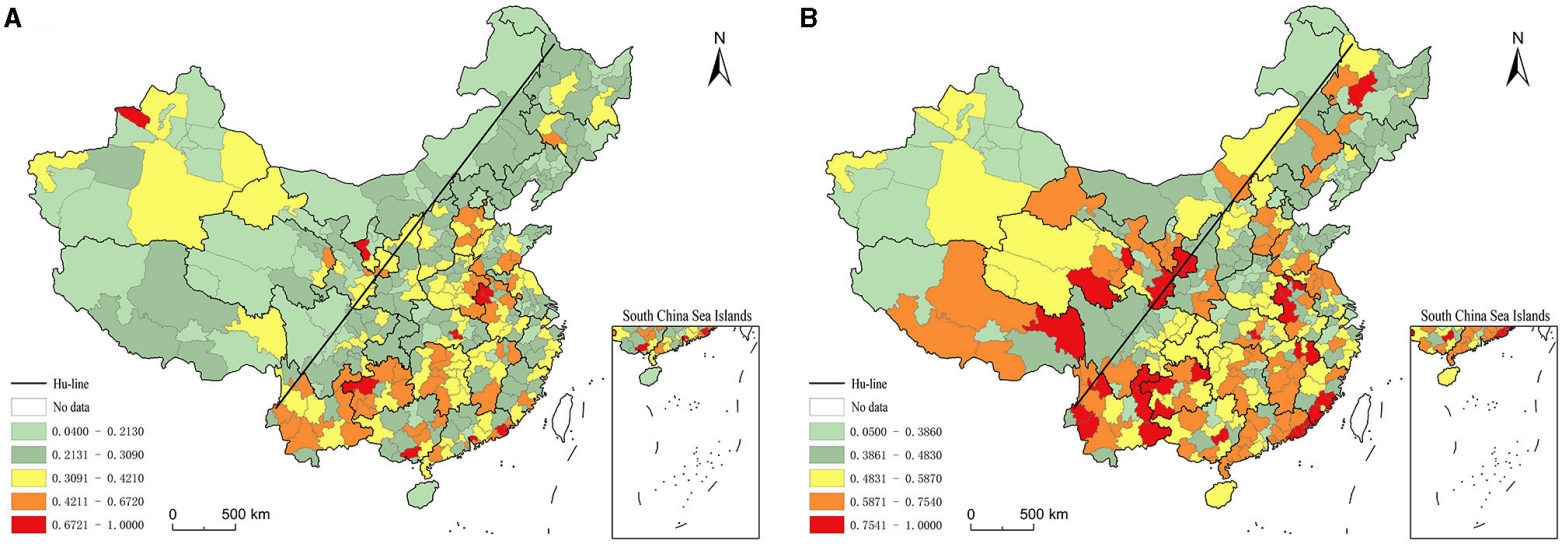
Distribution of production efficiency of health resources between prefectural districts in China. **(A)** 2000. **(B)** 2010.

In 2000, the high-value and second-highest-value areas of health productivity of health resources were mainly distributed in the regions of North China Plain, Yunnan-Guizhou Plateau and Southeast Coast. Among them, there were 11 high-value prefecture-level regions, including in Tianmen, Xiantao, Guyuan, Dongguan, Bortala, Bijie, Bozhou, Jieyang, Shantou, Fuyang, and Qinzhou. All of these regions have lower health resource inputs than the national average, with the exception of four places, Tianmen, Zhongwei, Bortala, Bijie and Qinzhou, all of which have higher health outputs than the national average. Sub-high-value areas 47, distributed in Yunnan and Guizhou, most of the coast of Guangdong, central Hunan, the border of Hubei, Anhui, Henan, and central Hebei and other regions. The higher health productivity in these areas is a direct result of either low health resource inputs and high health outputs, or low health resource inputs and low health outputs. Sub-low value area 128, low value area seven, mainly in the northwest, northeast and other regions, as well as Beijing, Shanghai, Tianjin, three municipalities directly under the Central Government and 15 provincial capital cities. Among them, in addition to Guangzhou, Taiyuan, Hohhot, Yinchuan, etc., the number of health institutions per 1,000 people is lower than the more national average, the other regions of the various health resources input are higher than the national average, while the health resources of health output in the western region at all levels of the region are significantly lower than the national average. Overall, high health resource inputs but no reciprocal improvement in health outputs are the direct cause of lower health productivity in some economically developed regions in the east, while higher inputs but lower health outputs are the direct cause of lower health productivity in some prefecture-level regions in the west. In 2000, the region-wide Moran index of health productivity of regional health resources at the prefecture level was 0.125, and passed the 0.01 significance level test, indicating a significant spatial clustering of its distribution. From the map of local spatial correlation index (Figure 2), it can be seen that the high and high concentration areas of health productivity are mainly concentrated in the whole area of Guizhou beyond the border of Sichuan, Yunnan and Guangxi, and the whole area of Guizhou outside Guiyang, coastal Guangdong, central Hunan, southern Jiangxi, and the border of Hubei, Anhui, Henan and Shandong, including 35 prefecture-level regions. The low-low catchment areas are mainly located in Xinjiang Hotan, Turpan, Changji and Altay inthe west, and Shenyang, Benxi, Dalian and Panjin in northeast China.

**Figure 3:**
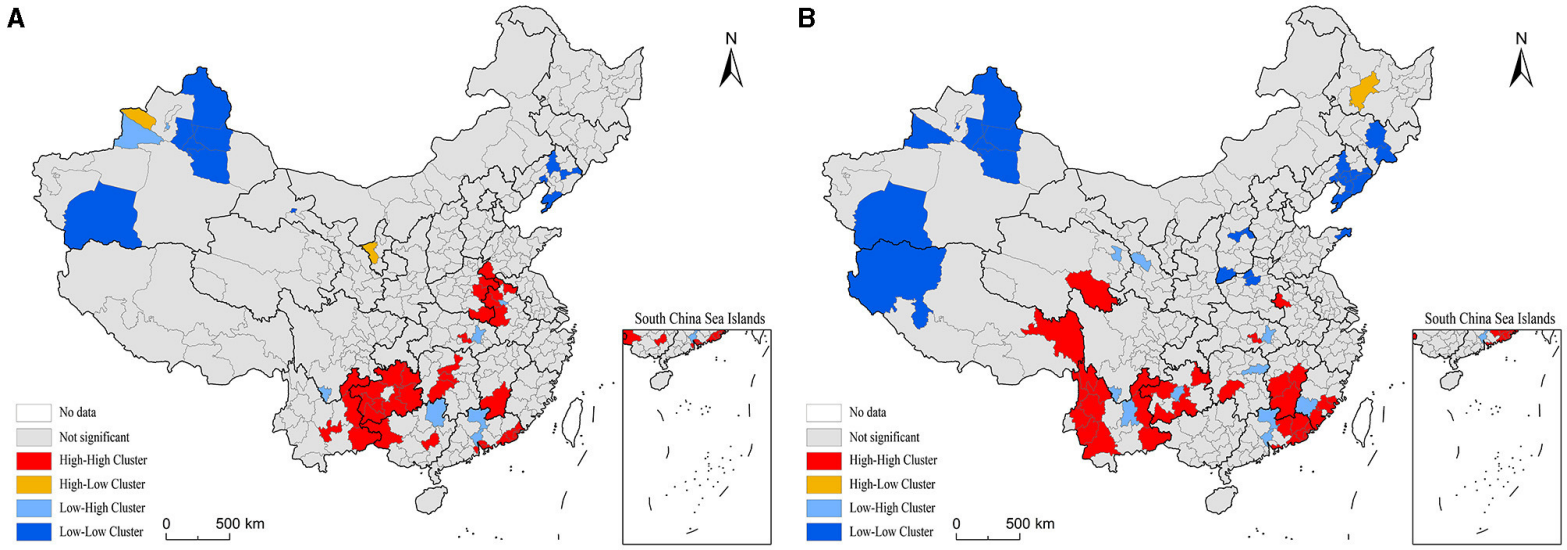
LISA cluster map of production efficiency of health resources between prefectural districts in China. **(A)** 2000. **(B)** 2010.

In 2010, there was a significant increase in high-value and sub-high-value areas and a significant decrease in low-value and sub-low-value areas for health resource health productivity (Figure 1). Among them, the number of high-value geographic areas increased to 32 and the number of sub-high-value areas increased to 72; while the number of sub-low-value geographic areas decreased to 93 and the number of low-value areas decreased to 59. High-value and sub-high-value areas show a trend of expansion and spread to the western and northeastern regions. Among them, the border of Yunnan, Guizhou and Sichuan, Henan and Anhui and the southeast coast are particularly obvious, and the areas of eastern Tibet, eastern Qinghai, southern Gansu, western Yunnan, southern Sichuan, southeastern Jiangxi and central northeast regions are added as high-value and sub-high-value contiguous areas. However, Beijing and 15 provincial capitals such as Guangzhou and Wuhan are still low value areas of health productivity. In general, the high value area and the second highest value area of health productivity are still basically characterized by low health resource input and high health output, or low health resource input and low health output. In contrast, the low-value and sub-low-value areas are basically characterized by high health resource inputs but no reciprocal improvement in health output, or even low health output. 2010 also saw a large change in the spatial association pattern of health productivity. The region-wide Moran index increased to 0.128, and passed the 0.01 significance level test, indicating a slight increase in spatial agglomeration; the coefficient of variation, on the other hand, plummeted compared with 2000, indicating a significant reduction in inter-regional differences and a significant increase in spatial dispersion. The local spatial association index map (Figure 3) clearly demonstrates the trend of changing the spatial pattern of health productivity of regional health resources at the prefecture level in China from concentration-clustering to decentralization-clustering. The regions of Changdu from western Yunnan to eastern Tibet, Gologu in Qinghai, and the border of Guangdong, Fujian, and Jiangxi were added as high-high concentration areas, but the high-high concentration areas in Guizhou, central Hunan, and the border of Anhui, Jiangsu, and Shandong contracted significantly. Northwest Henan in the central region, Taiyuan and Yangquan in Jinzhong were added as low-low agglomerations, while low-low agglomerations in the western and northeastern regions were expanded; the number of low-high agglomerations also increased significantly. In general, the spatial pattern of health productivity of regional health resources at the prefecture level in China shows the macroscopic characteristics of “general dispersion and local concentration”.

### 3.2 Factors influencing the health productivity of health resources

The Tobit model was implemented using Stata 15.0 software. Initially, all prefecture-level regions were considered as observational samples, and the model was fitted using the least squares method. The Hausman test statistic value of 278.12 with a *p*-value of 0.000 indicates that the original hypothesis is rejected at the 0.01 significance level, suggesting that the choice of the fixed effect estimation spatial panel Durbin model is superior. To further validate the robustness of the estimation results of the spatial panel Durbin model based on fixed effects, Wald and LR were used for robustness testing. The results show that the Wald and LR robustness of spatial lag and spatial error were both significant at the 0.01 level, indicating that the estimation results of the spatial panel Durbin model based on the fixed effects are well robust, and that the model cannot be simplified to Spatial Lag Model (SLM) and Spatial Error Model (SEM). In summary, a fixed-effects-based spatial panel Durbin Model (SDM) was used to estimate the spatial effects of the impact of each factor on the health productivity of health resources. The Lagrange multiplier test results indicated that both SEM and SLM were suitable, while the Hausman test suggested that a fixed-effects model was more appropriate. Consequently, a fixed-effects model akin to SDM was employed to analyze the factors influencing health resource productivity at the prefecture level in China.

Given the significant spatial heterogeneity observed using the Hu-line as a boundary, the model was separately applied to the eastern and western parts of the Hu-line to mitigate potential statistical confounding. The eastern model incorporated a total of 554 prefecture-level regions for the years 2000 and 2010, while the western model included 128 prefecture-level regions for the same period. The results of the model fitting are presented in Table 3, demonstrating a relatively good fit and allowing for the analysis of the direction and degree of influence of various factors on health resource productivity.

**Table 3:** Econometric test on influencing factors of production efficiency of health resources.

**Explanatory variables**	**National**	**East of the Hu-line**	**West of Hu–line**
ln *TL*	0.201^***^ (0.117)	0.151 (0.112)	0.332 (0.299)
ln *HA*	0.108^*^ (0.025)	0.152^*^ (0.028)	0.039 (0.055)
ln *HC*	−0.251^*^ (0.024)	−0.290^*^ (0.023)	−0.167^*^ (0.062)
ln *HP*	−0.153^*^ (0.050)	−0.115^**^ (0.051)	−0.106 (0.131)
ln *LT*	−0.029 (0.077)	0.163^*^ (0.084)	−0.339^**^ (0.157)
ln *LU*	0.040 (0.025)	0.029 (0.025)	0.112^***^ (0.065)
*HI*	−0.003 (0.003)	0.001 (0.005)	−0.003 (0.007)
*AP*	−0.021^**^ (0.009)	−0.023^*^ (0.009)	−0.025 (0.026)
*W*·ln *eff*	0.801^*^ (0.129)	0.654^*^ (0.209)	−1.169^*^ (0.519)
*W*·ln *TL*	−0.340 (1.112)	1.399 (0.942)	−5.145^**^ (2.230)
*W*·ln *HA*	−0.066 (0.221)	−0.851^*^ (0.209)	1.331^*^ (0.379)
*W*·ln *HC*	0.524^*^ (0.173)	0.551^*^ (0.150)	−0.764 (0.631)
*W*·ln *HP*	0.725^**^ (0.338)	1.031^*^ (0.300)	−0.113 (0.934)
*W*·ln *LT*	−0.027 (0.808)	−1.130 (0.708)	−2.689 (1.843)
*W*·ln *LU*	0.233 (0.437)	1.023^*^ (0.385)	−0.325 (0.724)
*W*·*HI*	−0.015 (0.016)	−0.030^***^ (0.016)	−0.030 (0.052)
*W*·*AP*	−0.138^***^ (0.080)	−0.007 (0.070)	−0.287^***^ (0.166)
Samples	682	554	128
log-likelihood	−134.282	−6.220	−52.807
*R* ^2^	0.719	0.787	0.391

Standard errors of regression coefficient estimates are in parentheses; ^*^, ^**^, and ^***^ indicate significant at the 1%, 5%, and 10% levels, respectively.

#### 3.2.1 Technical level of health institutions

The technical level of health institutions (ln*TL*) is generally positively correlated with the health productivity of regional health resources. Specifically, the regression coefficient of the technical level of health institutions in the national model is significantly positive. In both models east and west of the Hu-line, although the coefficients are not statistically significant, they are greater than zero, indicating a positive correlation between the technical level and health productivity. This suggests that an increase in the technical level of regional health institutions contributes to the enhancement of efficiency in health production of local health resources.

The estimated value of the regression coefficient for the spatial lag term (*W* ^*^ ln*TL*) of the technical level of health institutions reflects the influence of the technical level in neighboring regions on the health productivity of resources in the focal region. Overall, this variable has an overall negative effect on the efficiency of health production of local health resources. While the negative estimated value in the national model does not achieve significance, it still indicates a negative relationship between the technical level of health institutions in neighboring areas and the health productivity of health resources in the region. In the model east of the Hu-line, the correlation is non-significant, and in the model west of the Hu-line, it is significantly negative. Considering the results of both variables, it can be concluded that, holding other factors constant, the most effective strategy to enhance the health productivity of local health resources in each prefecture-level region, particularly those west of the Hu-line, is to actively improve the technical level of local health institutions.

#### 3.2.2 Accessibility of health services

The health service accessibility index (ln HA) is generally positively correlated with the health productivity of regional health resources. The regression coefficients of the health service accessibility index in the national model and the model east of the Hu-line are significantly positive. In the model west of the Hu-line, although not significant, the coefficients are greater than zero, indicating a positive correlation. This suggests that improving regional health service accessibility facilitates the enhancement of health productivity in local health resources. The estimated value of the regression coefficient of the spatial lag term (W ^*^ ln HA) of the health service accessibility index reflects the impact of the technical level of health institutions in neighboring regions on the health productivity of health resources in the region. There is significant spatial heterogeneity in the effect of this variable on the health productivity of regional health resources, with the variable being significantly positive in the model west of the Hu-line and the coefficient significantly negative in the model east of the Hu-line. A possible reason for this difference is that the poor accessibility of health services in prefecture-level regions west of the Hu-line, attributed to sparse population distribution and limited accessibility, may lead to an increase in health productivity. Improved accessibility in neighboring areas could attract local residents to seek care, resulting in improved health levels, even if local health resource inputs remain unchanged. Conversely, in prefecture-level areas east of the Hu-line with higher population density and accessibility, the increased accessibility of neighboring areas might attract residents for medical consultation. This could raise the burden on local health services, potentially leading to a crowding-out effect on local residents and a subsequent decrease in the efficiency of health production of local health resources.

The lnHC index was significantly and negatively correlated with the health productivity of regional health resources. This negative correlation between the index, reflecting the literacy level of regional residents, and health resource productivity indicates that the increase in residents' literacy is not accompanied by a rise in scientific awareness of health services. Consequently, health resource utilization decisions are not scientific, and excessive medical treatment may crowd out health resources, having a detrimental effect on normal health demand and leading to a decrease in health productivity of regional health resources.

The spatial lag term (*W* ^*^ ln*HC*) of the population health service perception index is generally positively related to the health productivity of local health resources. The estimated regression coefficient for this variable is significantly positive in the national model and the model east of the Hu-line; it is negative but not significant in the model west of the Hu-line. This suggests that the increase in the literacy level of the population in neighboring areas has a catalytic effect on the health productivity of local health resources.

The population health service affordability index (ln*HP*) is generally negatively correlated with the health productivity of regional health resources. The estimated regression coefficient for this variable is significantly negative in the national model and the model east of the Hu-line. In the model west of the Hu-line, the estimated regression coefficient, although not significant, is still less than zero, reflecting a negative correlation with health productivity. This suggests that the increase in residents' ability to pay for health services reduces the efficiency of local health resource production. The possible reason is that the increase in residents' ability to pay for health services, while enhancing the accessibility of health services, may also lead to overconsumption of health services and have a crowding-out effect on normal health demand, thus adversely affecting the efficiency of health production of local health resources.

The spatial lag term (*W* ^*^ ln*HP*) of the population health service affordability index is generally positively correlated with the health productivity of regional health resources. The estimated regression coefficient for this variable is significantly positive in the national model and in the model east of the Hu-line. It is negative but not significant in the model west of the Hu-line. This indicates that the increase in the ability to pay for health services of residents in neighboring areas has a catalytic effect on the health productivity of local health resources.

#### 3.2.3 Local public health policies

The medicalization index of health resource allocation (ln LT) generally exhibits a negative relationship with the health productivity of regional health resources, but this association displays spatial heterogeneity. Specifically, the estimated regression coefficient of this variable is significantly positive in the model east of the Hu-line, significantly negative in the model west of the Hu-line, and directly contributes to a negative but insignificant estimated regression coefficient of this variable in the national model. This indicates that nationally, there is a certain issue with health resource allocation, emphasizing medical treatment over prevention, which negatively impacts the health output of regional health resources. Particularly in the prefecture-level regions west of the Hu-line, the problem of an unreasonable health resource allocation structure is more severe, and the lack of prevention resources reduces the overall health output level of health resources. In the prefecture-level regions east of the Hu-line, with a larger population size, higher population density, and greater healthcare needs, there is a higher allocation of medical resources, contributing to improved health output. The correlation between the spatially lagged term of the medicalization index (*W* ^*^ ln *LT*) and the health productivity of regional health resources was not spatially heterogeneous, and although it did not pass the significance test, the estimated regression coefficients of this variable were negative in all three models, still indicating to some extent that an increase in the medicalization of health resource allocation in neighboring regions can adversely affect the level of health output of local health resources. Combining these results, it can be inferred that the overall health output level of health resources can be improved by placing more emphasis on the allocation of preventive health resources in all regions, especially in the western region, and by balancing medical treatment and prevention.

The urbanization index of health resource allocation (ln *LU*) is generally positively related to the health productivity of regional health resources. Among them, the regression coefficient estimates of this variable are significantly positive in the model west of the Hu-line. In the national model and the model east of the Hu-line, although the coefficient estimates of this variable do not pass the significance test, the results greater than zero reflect the positive correlation dynamics between the two. This may be due to municipal districts in each district-level region being centers of population concentration with high demand for health resources. Although the supply of health resources is tilted and concentrated in the municipal districts, the supply has not yet exceeded the demand, and the health output of health resources thus remains in the improvement zone. Especially in the western region, the contribution of increased municipal health resource allocation to overall regional health output was more pronounced.

The spatial lag term of the urbanization index of health resource allocation (*W* ^*^ ln *LU*) was generally positively correlated with the health productivity of regional health resources. Among them, the estimated regression coefficient of this variable is significantly positive in the model east of the Hu-line; it is negative but not significant in the model west of the Hu-line. This suggests that the increased urbanization of health resource allocation in neighboring areas has a catalytic effect on the health productivity of local health resources. The possible reason for this is that a higher level of health care services in neighboring municipalities will attract local residents to enter for medical care, thus improving the health of local residents, which, in turn, will have a positive impact on the health productivity of local health resources.

#### 3.2.4 Regional natural environment quality

The temperature and humidity index (HI) is generally negatively correlated with the health productivity of regional health resources. Among them, the estimated regression coefficients of the temperature and humidity index in the national model and the model west of the Hu-line are negative, although not significant, still reflecting a negative correlation with health productivity. In the model east of the Hu-line, the coefficient of this variable is positive but not significant. The degree of climate suitability is generally higher in the East, so fluctuations in the temperature and humidity index have little effect on the level of health output from health resources. The spatial lag term (W ^*^ HI) of the temperature and humidity index was negatively correlated with the health productivity of regional health resources, especially in the region east of the Hu-line, and the negative correlation was significant, indicating that the reduced climate suitability of neighboring regions would have a negative impact on the health output of local health resources. Taken together, the above results suggest that regional climate suitability is beneficial to the level of health output of health resources in all regions.

The air pollution index (*AP*) is negatively related to the health productivity of health resources in the region. Among them, the regression coefficient estimates of the air pollution index are significantly negative in the national model and the model east of the Hu-line; in the model west of the Hu-line, the negative results of the coefficient estimates of this variable reflect the negative correlation dynamics between air pollution and health output levels of health resources in the region, although they do not pass the significance test. The spatial lag term (*W*·*AP*) of the air pollution index is also negatively related to the health productivity of regional health resources. Among them, the coefficient estimates of the spatial lag term of the air pollution index were significantly negative in the national model and the model west of the Hu-line, and the coefficient estimates of this variable were negative but did not pass the significance test in the model east of the Hu-line. Taken together, these results suggest that regional air quality improvement is beneficial to the health productivity of health resources in all regions.

## 4 Discussion

### 4.1 In terms of spatio-temporal pattern

In this paper, the spatial scale of China's health resource health production efficiency research was expanded from the national and provincial scales to smaller spatial scales, and for the first time, it was extended to the municipal scale, and a comparative study of the regional scale was carried out, which fully takes into account the impact of the internal spatial heterogeneity on the results of the study, and the data were more detailed, and the conclusions of the study were more scientific and persuasive. The overall pattern of health productivity of health resources in China has obvious regional differences of “high in the south-east and low in the north-west”, which was similar to the pattern of China's economic development in which the east was strong and the west was weak, reflecting the role of economic development in promoting the health productivity of health resources in the region. However, according to the conclusion of Chen and Zhu's (44) study on the gradual incremental increase of China's regional economic spatial differences from the coast to the inland during the same period of time, the spatio-temporal pattern of China's health resource health productivity was again very different from the pattern of regional economic differences and the results of their changes, and therefore the level of health resource health productivity cannot be judged simply by the level of regional economic development.

### 4.2 In terms of influencing factors

An analysis of the factors influencing the health production efficiency of health resources in China reveals that the technical level of health institutions, accessibility of health services, local public health policies, and regional natural environment quality all significantly influence the health production efficiency of regional health resources, and were characterized by complexity, diversity, and differentiation. Comprehensively grasping the differences in the factors affecting the health productivity of health resources, promoting the expansion of research to the community and other micro scales, digging deeper into the mechanisms affecting the health productivity of health resources at the micro scale, and taking into account the combined effects of the influencing factors at the micro and macro scales will help to configure interventions in a more precise way and achieve spatial equilibrium in the productivity of health at the regional level.

### 4.3 Policy suggestions

In summary, the health productivity of regional health resources was the result of the combined effect of multiple factors. The level of regional economic development and the quality of ecological environment, the structure of health resources supply, the quality of health care system operation, the residents' health resources utilization decision and health service purchasing ability all affect the health output level of health resources. Possible ways to increase the health productivity of health resources include: optimizing the structure of health resource supply factors, with equal emphasis on prevention and treatment; optimizing the spatial structure of health resource supply, expanding the supply of resources in population catchment areas; optimizing the human capital structure of the health care system, and improving the technical level of health care services; Promote the fair distribution of income, optimize the transportation system, and improve the accessibility of regional health services; guide residents to correctly understand health care and medical and health services, and improve the scientific nature of health resource utilization decisions; strengthen ecological and environmental management, and improve the quality of the regional ecological environment.

### 4.4 Limitations and future research

There are several improvements in this study. In terms of health resource health productivity measures, limited by the availability of data, regional health resource input factors are not considered comprehensively, especially financial inputs are not considered enough; Health output indicators are mainly macro and long time scale indicators, and there is a lack of more micro immediate indicators that can directly reflect the health care system to improve the health of the population. With regard to the factors influencing the health productivity of regional health resources, in-depth research is needed on the transmission mechanism of neighboring regional factors affecting the health output level of local health resources. In terms of the time scale of the empirical analysis, it is expected that the 7th National Census sub-county data will be obtained as soon as possible to extend the study period to ensure the timeliness of the study.

## 5 Conclusions

There are evident regional disparities in the health productivity of health resources in China, notably divided by the Hu-line, where the spatial heterogeneity follows a pattern of “high in the southeast and low in the northwest.” While regional differences decreased significantly between 2000 and 2010, there was a slight increase in spatial correlation. This shift suggests a transition in the spatial pattern of health productivity at the prefecture level, moving from centralized-collective to decentralized-collective, ultimately forming a macroscopic feature characterized by “overall decentralization and localized clustering.”

The results of the spatial Durbin model revealed various factors influencing the health productivity of regional health resources. The technical level of local health institutions exhibited a positive impact, while the technical level of health institutions in neighboring areas had a negative effect. Local health service accessibility positively influenced health resource productivity, but spatial heterogeneity was observed in the impact of health service accessibility in neighboring areas, with a negative effect in regions east of the Hu-line and a positive effect in regions west of the Hu-line.

The literacy level of local residents had a negative effect on health resource productivity, whereas neighborhood residents' literacy exhibited a positive impact. Local residents' ability to pay for health services negatively affected health resource productivity, while neighborhood residents' ability to pay for health services had a generally positive effect.

The degree of medicalization in local health resource allocation had spatially heterogeneous effects on health resource productivity, with a positive impact in regions east of the Hu-line and a negative impact in regions west of the Hu-line. Additionally, the degree of medicalization in health resource allocation in neighboring regions negatively influenced the health productivity of health resources.

The urbanization level of health resource allocation in both local and neighboring areas generally had a positive effect on health resource productivity. Climate suitability in both local and neighboring areas positively influenced health resource productivity, whereas air pollution in both local and neighboring areas had a negative impact on health resource productivity.

## Data availability statement

The datasets presented in this study can be found in online repositories. The names of the repository/repositories and accession number(s) can be found in the article/supplementary material.

## Author contributions

HT: Data curation, Formal analysis, Methodology, Software, Visualization, Writing – original draft, Writing – review & editing. RA: Conceptualization, Investigation, Writing – review & editing. YL: Resources, Validation, Writing – review & editing.
